# Icariin, an Anti-atherosclerotic Drug from Chinese Medicinal Herb Horny Goat Weed

**DOI:** 10.3389/fphar.2017.00734

**Published:** 2017-10-12

**Authors:** Jian Fang, Yongjun Zhang

**Affiliations:** ^1^Department of Pharmacy, Huadu District People’s Hospital, Southern Medical University, Guangzhou, China; ^2^Department of Gastroenterology, Huadu District People’s Hospital, Southern Medical University, Guangzhou, China

**Keywords:** icariin, atherosclerosis, endothelial function, foam cell, smooth muscle cell

## Abstract

Icariin is a major bioactive pharmaceutical constituent isolated from Chinese medicine Horny Goat Weed (Ying Yang Huo) with potent cardiovascular protective functions. Emerging evidence in the past decade has shown that Icariin possesses multiple atheroprotective functions, through multiple mechanisms, including attenuating DNA damage, correcting endothelial dysfunction, inhibiting the proliferation and migration of smooth muscle cells, repressing macrophage-derived foam cell formation and inflammatory responses, as well as preventing platelet activation. All of these protective effects, combined with its lipid-modulatory effects, contribute to the broad atheroprotective effects of Icariin. In this review, we will summarize the anti-atherosclerotic properties of Icariin and highlight future perspectives in developing Icariin as a promising anti-atherosclerotic drug.

## Introduction

Cardiovascular diseases (CVDs) represent one of the leading causes of death in the modern society worldwide (Benjamin et al., 2017). At present, due to the complexity of the pathogenesis of atherosclerosis, therapeutic drugs that can prevent or treat atherosclerosis remain very limited (Orekhov et al., 2015). Currently, in light of the fact that atherosclerosis is a lipid disorder; lipid-lowering statins are the main category of anti-atherosclerotic drugs. Recently, several new therapeutic options are showing promising prospect in treating atherosclerosis, such as PCSK9 monoclonal antibodies (Pedersen, 2016). Despite the wide application of statins, the mortality rate of CVD remains very high, suggesting the need to develop new drugs that can treat atherosclerosis.

Numerous studies have shown that atherosclerosis is a chronic inflammatory disease with the characteristics of persistent inflammatory response, inflammasome activation, and impaired inflammation resolution (Ross, 1993; Rader and Daugherty, 2008; Libby et al., 2011; Tabas et al., 2015). Many vascular cells, including endothelial cells, smooth muscle cells, monocytes, macrophages, and platelets are involved in the inflammatory responses of vessel wall in response to pro-atherogenic stimuli (Rader and Daugherty, 2008). It has been reported that the current “gold standard” anti-atherosclerotic drugs such as statins can also produce anti-inflammatory effects independent of lipid-lowering effects (Rosenson, 2004). Therefore, anti-inflammatory therapy is a good strategy for the prevention and treatment of atherosclerosis. As we are entering a golden era of developing drugs from natural products (Shen, 2015), natural products especially Chinese medicinal herbs emerges as important sources of cardiovascular protective medicine that target multiple cellular processes of atherosclerosis. These natural products would probably translate into effective novel cardiovascular therapeutics (Amirkia and Heinrich, 2015; Fang et al., 2017).

**FIGURE 1:**
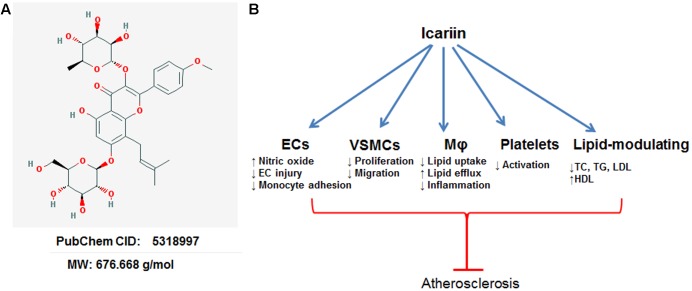
Anti-atherosclerotic effects of Icariin. EC, endothelial cell; VSMC, vascular smooth muscle cell; Mφ, macrophage. **(A)** The chemical structure of Icariin obtained from PubChem database. **(B)** Atheroprotective effects of Icariin.

Horny Goat Weed, an eminent Chinese herbal medicine (also known as Epimedii Herba or Ying Yang Huo), is widely used to treat various diseases such as coronary heart disease, impotence and osteoporosis, and rheumatism (Ye and Chen, 2001). Among the pharmacologically active constituents, Icariin (**Figure 1A**) is a major bioactive pharmaceutical constituent that has been reported to treat multiple CVDs (He et al., 1995; Xu and Huang, 2007) via anti-oxidant, anti-inflammatory, and lipid-modulatory effects. The excellent cardiovascular protective profiles of Icariin make it an excellent drug candidate for cardiovascular therapeutics. In the following sections, we will present an overview of the cardiovascular protective effects and molecular mechanisms of Icariin in atherosclerosis.

## Anti-Atherosclerotic Effects of Icariin

Previous studies have suggested that Icariin attenuated the development of atherosclerosis in several animal models, including ApoE^-/-^ mice (Xiao et al., 2015, 2017; Wang et al., 2016), rats (Hu et al., 2015b), and rabbits (Zhang et al., 2013). The atheroprotective mechanisms of Icariin are exerted through anti-oxidative, anti-inflammatory, anti-thrombotic, and lipid-modulatory effects. Cellular models are very useful in evaluating the therapeutic effects of disease-modulating drugs in cultured cell systems (Orekhov et al., 2015). Icariin has demonstrated eminent protects against atherosclerosis in multiple cellular models, including: endothelial dysfunction, macrophage-derived foam cell formation, aberrant proliferation and migration of smooth muscle cells and platelet activation (**Figure 1B**).

### Anti-DNA Damage Effects

The generation of reactive oxygen species (ROS) contributes to the development of many diseases, including atherosclerosis. Icariin has polyphenol structure which shares similar antioxidant properties as other polyphenols. Liu et al. (2004) evaluated the antioxidative effects of icariin on free-radical [AAPH, 2,2′-azobis(2-amidinopropane hydrochloride)]-induced hemolysis of erythrocytes. The authors observed that Icariin has protective effects against free-radical-induced peroxidation in erythrocytes, although much weaker than its structural analogs norwogonin and acacetin. Zhao et al. (2007) followed to examine the anti-oxidant effects of Icariin in a cell-free system and the authors found that Icariin reduced AAPH-induced DNA damage. The observed anti-DNA damage effects could contribute to the reduced DNA damage marker γ-H2AX expression and lifespan-extension effect by Icariin in C57BL/6J mice (Zhang et al., 2015; Shen et al., 2017).

### Preventing Endothelial Dysfunction

The endothelium is important in keeping the health and function of the vessel since it is the direct cell type that closely contacts the circulating blood. Main functions of endothelium include the regulation of vascular tone, barrier function, anti-oxidative, anti-inflammatory, and anti-thrombotic status (Gimbrone and Garcia-Cardena, 2016). However, endothelial function impairment by pathological stimuli causes endothelial dysfunction, an initiating event in the development of atherosclerosis. Endothelial dysfunction is a complex and broad term that encompass several aspects, such as impaired NO production, eNOS uncoupling, monocyte adhesion to endothelial cells, endothelial senescence, and apoptosis, etc. (Xu et al., 2016b). In terms of NO production, endothelium is the major cell type that produces NO through the endothelium nitric synthase (eNOS) pathway. In this regard, Icariin protects against H_2_O_2_ induced apoptosis in endothelial cell line ECV304 by decreasing the activation of caspase 3 and boosting the production of vasodilatory NO derived from eNOS (Wang and Huang, 2005). Further studies showed that Icariin increases the production of NO by activating PI3K/pAkt/p-eNOS (S1177) and EGF/EGFR pathways (Chung et al., 2008; Liu et al., 2011). In agreement with this finding, another study congruently shows that androgen receptor (AR), PI3K/Akt, and ERK pathway are involved in increased release of NO via eNOS activation (Koizumi et al., 2010). Also, through the Akt/eNOS/NO pathway, Icariin prevents endothelial senescence induced by homocyteine both *in vitro* and *in vivo* (Xiao-Hong et al., 2013) as well as *s*-nitrosogultathione induced endothelial apoptosis (Huang et al., 2016). In addition to the upregulation of eNOS phosphorylation at Ser1177, Icariin also increases eNOS mRNA and protein expression, suggesting Icariin could also regulate eNOS level at the transcriptional and translational level (Xu and Huang, 2007). Furthermore, Icariin also increase eNOS activity by blocking eNOS uncoupling through decreasing the level of ROS and asymmetric dimethylarginine (ADMA) by increasing the level of dimethylarginine dimethylaminohydrolase (DDAH) in ApoE^-/-^ mice. The resulted effect is increasing endothelium-dependent vasorelaxation (Xiao et al., 2015). Recent evidence has also suggested that Icariin reduces oxLDL-induced monocyte adhesion to endothelial cells, the initiating event in atherosclerosis. The mechanism is linked to decreasing the expression of pro-adhesive cytokines ICAM-1, VCAM-1 and E-selectin, probably through NF-κB pathway (Hu et al., 2015a). In terms of the important role of SIRT6 in regulating endothelial dysfunction and atherosclerosis (Liu et al., 2016; Xu et al., 2016a,c; Zhang et al., 2016), Icariin has recently been reported as a SIRT6 pharmacological activator *in vitro* and *in vivo* (Chen et al., 2015), pinpointing the potential contribution of SIRT6 activation to the atheroprotective effects of Icarrin. The endothelial protective effects of Icariin contributed to upregulation of eNOS-dependent NO production in ApoE^-/-^ mice fed a high fat diet (Xiao et al., 2017).

### Inhibiting the Proliferation and Migration of Smooth Muscle Cells

After endothelial injury, the vascular smooth muscle cells (VSMCs) will migrate from the media layer to the sub-endothelial space and proliferate very quickly, forming the neointima, the early form of vascular remodeling associated with atherosclerosis. Markers for VSMC proliferation include proliferating cell nuclear antigen (PCNA), Ki67 and cell cycle progression. A recent study by Hu colleagues has shown that Icariin dose-dependently (10–40 μM) suppressed oxidized LDL induced proliferation of VSMCs by inhibiting the ERK1/2 pathway and PCNA expression. Icariin treatment also arrests cell cycle at S and G2/M phase (Hu et al., 2016). Accelerating the death of lesional smooth muscle cells is another way of inhibiting the proliferation and migration of VSMCs. In this regards, Icariin decreases VSMC turnover by promoting the apoptosis through glucose regulated protein 78 (GRP78) in homocysteine-treated VSMCs (Shen and He, 2009). All these effects contribute to Icariin-mediated anti-proliferative effects in VSMCs.

### Inhibiting Inflammation and Macrophage-Derived Foam Cell Formation

Inflammation is a key process involved in all phases of atherosclerosis (Ross, 1993; Rader and Daugherty, 2008; Libby et al., 2011; Xu et al., 2014a,b; Tabas et al., 2015). Icariin has been shown to inhibit inflammation in lipopolysaccharide (LPS)-stimulated macrophages by suppressing the expression of TNFα, COX2, iNOS, and MPO activity (Chen et al., 2010; Xu et al., 2010) through activating PI3K/Akt pathway and inhibiting p38 MAPK and NF-κB p65 pathways. Transcriptome profiling is a useful method to dissect the molecular mechanisms of pharmaceutical compounds (Xu, 2017). By using transcriptomic profiling, a recent study has shown that Icariin reduces the expression of pro-migratory chemokine CX3CR1 in LPS-stimulated macrophages as well as CX3CL1 induced macrophage chemotaxis (Wang et al., 2016). As a result, Icariin significantly reduces macrophage infiltration in aortic plaques from ApoE^-/-^ mice fed a high fat diet (Wang et al., 2016).

In addition to inflammation in macrophage, macrophage-derived foam cell formation is the hallmark of atherosclerosis. It is a complex cellular event determined by the balance of cholesterol uptake and efflux (Liu et al., 2015). Both cholesterol uptake and efflux are mediated by several scavenger receptors and transporters. Specifically, cholesterol uptake is mediated by CD36, SRA and LOX-1, whole efflux is mediated by ABCA1, ABCG1, and SRBI (Xu et al., 2013). The intracellular level of cholesterol is determined by the net effect of both processes. In this regard, Icariin has been shown to reduce CD36 expression and upregulate SRBI expression through p38 MAPK pathway in macrophages stimulated with LPS and oxidized LDL (Yang et al., 2015).

### Icariin Modulates Lipid Metabolism

In a rat model of atherosclerosis by feeding a high cholesterol diet and Vitamin D3, 4 weeks of treatment with Icariin (30 and 60 mg/kg/d, intragastric administration) significantly decreased the levels of TC, TG, LDL-C, while increase that of HDL-C (Hu et al., 2015b). In ApoE^-/-^ mice fed a high fat diet, Icariin reduces atherosclerosis by reducing the concentrations of TC and TG (Xiao et al., 2017). Icariin also reduced the levels of serum TC and LDL-C, as well as the atherosclerotic burden in rabbits (Zhang et al., 2013). These findings suggest the broad lipid-modulatory effects of Icariin are accountable for its atheroprotective effects observed in animal models.

### Inhibiting Platelet Activation

Platelet activation and ensuing thrombus formation is a key event in the late stage of atherosclerosis, and drugs that inhibit platelet activation are promising candidates of treating atherosclerosis. Icariin has been found to improve the imbalance between plasminogen activator inhibitor-1 (PAI-1) and tissue-type plasminogen activator (t-PA) activities, thereby reducing platelet activation in hypercholesterolemia in a rabbit model of atherosclerosis (Zhang et al., 2013).

## Potential Molecular Targets of Icariin

**FIGURE 2:**
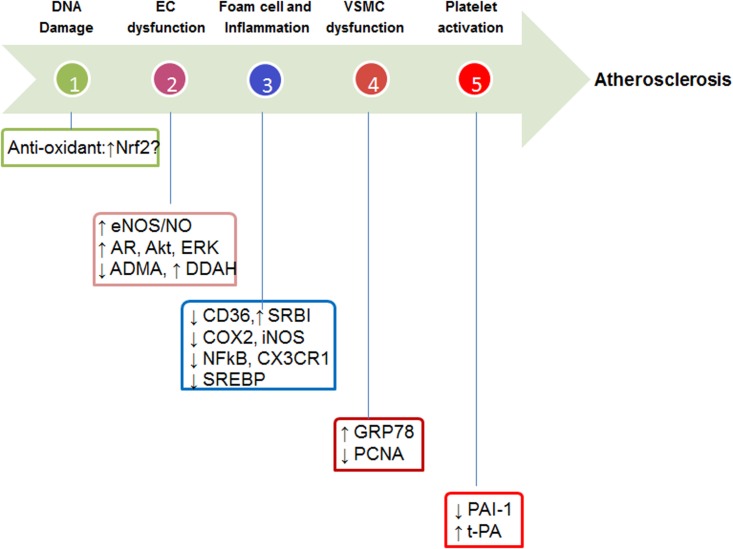
Potential molecular targets of Icariin. Nrf2, eNOS, endothelial nitric oxide synthase; NO, nitric oxide; AR, androgen receptor; ADMA, asymmetric dimethylarginine; DDAH, dimethylarginine dimethylaminohydrolase; CD36, cluster of differentiation 36; SR-BI, scavenger receptor B1; COX2, cyclooxygenase 2; iNOS, inducible NO synthase; NF-κB, nuclear factor-kappa B; CX3CR1, SREBP, sterol regulatory element-binding protein; GRP78, glucose regulated protein 78; PCNA, proliferating cell nuclear antigen; PAI-1, plasminogen activator inhibitor-1; t-PA, tissue-type plasminogen activator.

Icariin has potent anti-oxidative, anti-inflammatory, anti-proliferative, anti-thrombotic, and lipid-modulatory effects in vascular cells. The potential molecular targets are summarized in **Figure 2**. (i) Molecular targets of anti-oxidant effects: icariin has direct free-radical scavenging effects, which constitute the basis for its preventive effects against DNA damage. Another potential anti-oxidant mechanism could be the activation of Nrf2 in light of the recent observation (Wu et al., 2014) showing that Icariin derivative Icaritin activates Nrf2 in cigarette smoke extract treated-human lung epithelial cells. Further studies are needed to support the Nrf2 activation effects of Icariin in vascular cells; (ii) molecular targets of anti-inflammatory effects: inflammation in vascular cells, macrophage in particular, is mainly driven by transcriptional factor NF-κB (Wu et al., 2010). Icariin has been demonstrated to reduce the expression of COX2, iNOS, and CX3CR1 (Chen et al., 2010; Xu et al., 2010), which are NF-κB direct target genes. Therefore, inhibition of NF-κB could be the primary target of Icariin-mediated anti-inflammatory effects; (iii) molecular targets of anti-proliferative effects: the anti-proliferative targets of Icariin are PCNA (Hu et al., 2016) and GRP78 (Shen and He, 2009); (iv) molecular targets of anti-thrombotic effects: icarrin exhibits protective effects against platelet activation, and the molecular targets are pro-thrombotic PAI-1 and anti-thrombotic t-PA (Zhang et al., 2013); (v) molecular targets of lipid-modulatory effects: the molecular mechanisms of Icariin in modulating lipid metabolism has not been fully explored. Previous studies in experimental animal models have suggested that Icariin reduces bad LDL-cholesterol, while increase good HDL-cholesterol (Hu et al., 2015b). The potential mechanisms could be twofold. On the one hand, Icariin could inhibit cholesterol synthesis and uptake via sterol regulatory element-binding protein (SREBP) (Han et al., 2016) and scavenger receptor (CD36) (Yang et al., 2015), respectively. On the other hand, Icariin could promote SR-BI-mediated cholesterol efflux (Yang et al., 2015) and facilitate cholesterol excretion. The potential effects of Icariin on HDL biogenesis warrant further studies.

## Concluding Remarks and Future Perspective

Emerging evidence from the past several years has indicated that Icariin is a promising naturally occurring compound that possesses multiple atheroprotective functions. The molecular mechanism involves anti-inflammatory, anti-oxidative and lipid-modulatory effects. Since Icariin is identified as a PDE5 inhibitor (Xin et al., 2003) and SIRT6 activator (Chen et al., 2015), it remains to be investigated in future whether the atheroprotective actions of Icariin are dependent on PDE5 inhibition and SIRT6 activation. There are also several aspects that need to be investigated in future studies: (1) the effect of Icariin on VSMC plasticity, i.e., the phenotypic switch from contractile to synthetic phenotype; (2) the effect of Icariin on macrophage transmigration to endothelium, the key initiating events in atherosclerosis; (3) the effect of Icariin on macrophage polarization, i.e., M1 to M2 switch; (4) Icariin has broad lipid-modulatory effects, while, the effects of Icariin on hepatic cholesterol synthesis and metabolism remains to be determined; (5) Icariin derivatives has showed potent anti-inflammatory effects in macrophages (Chen et al., 2010), it is also important to design and test the therapeutic efficacy of Icariin derivatives in treating atherosclerosis in experimental models. With the help from biotechnological advances such as RNA-sequencing, transcriptomic studies in Icariin treated vascular cells under disease settings will help identify novel therapeutic targets of Icariin. Further understanding of mechanism of action of Icariin will help us gain a comprehensive understanding of the anti-atherosclerotic profile of Icariin.

## Author Contributions

JF and YZ contributed to the conception, drafting, and revision of the manuscript and approved the final version.

## Conflict of Interest Statement

The authors declare that the research was conducted in the absence of any commercial or financial relationships that could be construed as a potential conflict of interest.
